# A non-invasive method to determine core temperature for cats and dogs using surface temperatures based on machine learning

**DOI:** 10.1186/s12917-024-04063-2

**Published:** 2024-05-14

**Authors:** Zimu Zhao, Xujia Li, Yan Zhuang, Fan Li, Weijia Wang, Qing Wang, Song Su, Jiayu Huang, Yong Tang

**Affiliations:** 1 School of Information and Software Engineering, University of Electronic Science and Technology of China, Chengdu, China; 2 Center for Artificial Intelligence in Medicine, The Affiliated Hospital of Southwest Medical University, Luzhou, China; 3 Department of General Surgery (Hepatobiliary Surgery), The Affiliated Hospital of Southwest Medical University, Luzhou, China; 4 College of Computer Science, Sichuan University, Chengdu, China; 5 College of Blockchain Technology, Chengdu University of Information Technology, Chengdu, China; 6 Genesis AI Lab, Futong Technology, Chengdu, China; 7 Xinwang Animal Hospital, Luzhou, China; 8 School of Computer Science and Engineering, University of Electronic Science and Technology of China, Chengdu, China

**Keywords:** Companion animal, Cat, Dog, Core temperature, Machine learning

## Abstract

**Background:**

Rectal temperature (RT) is an important index of core temperature, which has guiding significance for the diagnosis and treatment of pet diseases.

**Objectives:**

Development and evaluation of an alternative method based on machine learning to determine the core temperatures of cats and dogs using surface temperatures.

**Animals:**

200 cats and 200 dogs treated between March 2022 and May 2022.

**Methods:**

A group of cats and dogs were included in this study. The core temperatures and surface body temperatures were measured. Multiple machine learning methods were trained using a cross-validation approach and evaluated in one retrospective testing set and one prospective testing set.

**Results:**

The machine learning models could achieve promising performance in predicting the core temperatures of cats and dogs using surface temperatures. The root mean square errors (RMSE) were 0.25 and 0.15 for cats and dogs in the retrospective testing set, and 0.15 and 0.14 in the prospective testing set.

**Conclusion:**

The machine learning model could accurately predict core temperatures for companion animals of cats and dogs using easily obtained body surface temperatures.

## Background

Pet ownership is common in modern society, with cats and dogs being the most commonly owned pets [1]. According to a recent report, more than 88 million European households (38% of all households) had at least one pet in 2021 [2]. Some studies suggested that human-companion animal ownership or interactions may help improve the overall quality of life, including physical, social, and mental health [3–5]. However, the increasing prevalence of animal diseases has elevated the risk and has a detrimental impact on animal welfare [6]. Moreover, Zoonosis such as dengue fever, hepatitis E, toxoplasmosis, etc., make the companion animal has the potential for human pathogenicity [7]. Therefore, attention needs to be paid to the health status of animals for prompt diagnosis and treatment.

The change of the body temperature of an animal is closely related to the diagnosis and treatment of diseases [8]. Clinically, rectal temperature (RT) is an important index of core temperature, which has guiding significance for the diagnosis and treatment of pet diseases [9, 10]. However, this invasive approach is not tolerated in all cats and dogs [11]. In addition, due to the influence of intestinal air, feces, and lumps, RT measured by this method is slightly lower and often lags behind the temperature changes. For these reasons, alternative methods such as infrared ear thermometer, axillary temperature record, and infrared thermogram were used to estimate the rectal temperature [11, 12]. However, these alternatives were found inaccurate in predicting RT [12–15], which are difficult to meet clinical needs. Therefore, it is necessary to find a simple, fast, and accurate method of determining pet core temperatures.

Recent years witnessed significant advances in artificial intelligence (AI) with groundbreaking successful applications in a range of domains [16]. Machine learning (ML) is a branch of AI, consisting of various mathematical methodologies to conduct tasks like classifications, regressions, dimensionality reductions, and density estimations [17]. In a typical machine learning process, predictive models were trained based on the available datasets to obtain generalization, namely the capability to accurately process unseen or future datasets [18]. There were abundant reports using machine learning in human medicine [17]. More recently, machine learning was found in zoology. Especially, there were emerging studies applying machine learning to animal health. Renard et al. developed an algorithm to predict the short-term and medium-term survival rates of cats with acute and chronic kidney diseases [19]. Banzato et al. used a convolutional neural network (CNN) to identify common radiological findings from chest X-rays in cats [20]. Vehkaoja et al. proposed a machine learning algorithm for dog behavior recognition and classification [21]. We noticed that there was literature investigating approaches to determine core temperatures in the human body [22] and economical animals like rabbits and piglets [23, 24]. However, the usefulness of machine learning models to predict core temperatures of companion animals like cats and dogs are unclear.

Therefore, in this study, we proposed an alternative method based on machine learning to replace direct measuring RT for cats and dogs. We systematically developed and evaluated a core temperature predictive model using the measurements of surface temperatures of body areas. The machine learning based model was developed and evaluated in a retrospective dataset and a prospective dataset, respectively. The results showed that the model could achieve satisfying accuracies in predicting core temperatures of cats and dogs using measurements of the nasal, ear, oral cavity, abdomen, axillary, perianal, core, and room temperatures.

## Methods

### Data collection

**Fig. 1:**
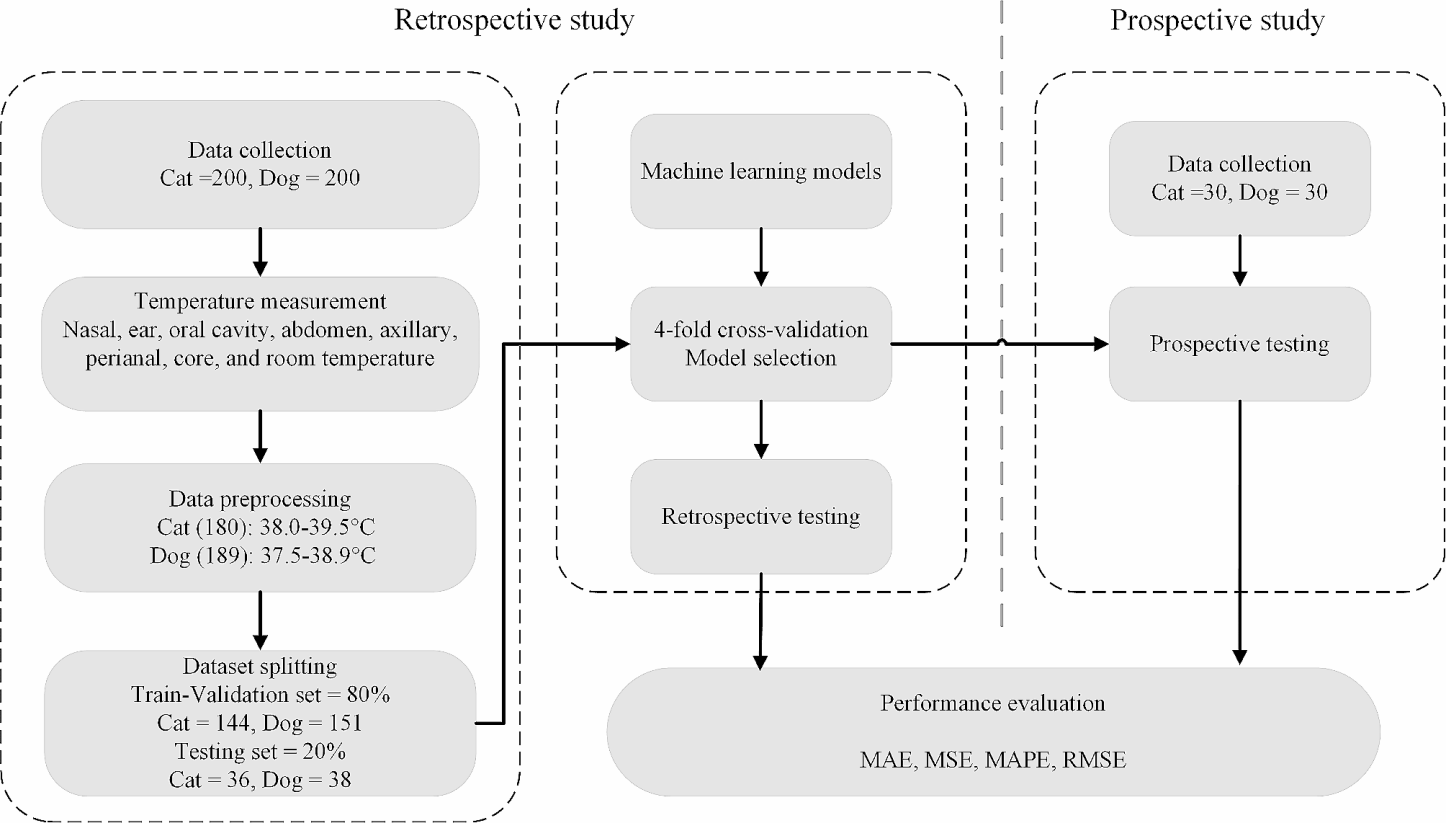
Flowchart of data collection, model development and evaluation in the retrospective and prospective studies. MAE: mean absolute error, MSE: mean square error, MAPE: mean absolute percentage error, RMSE: root mean squared error

In this study, we conducted both retrospective and prospective studies. As illustrated in Fig. 1, we first retrospectively collected data from four clinics that are part of Xinwang Animal Hospital. The inclusion criteria were companion cats and dogs that were admitted to the Xinwang Animal Hospital and tolerated to body temperature measurements. All temperature measurements were made with the consent of the owners. Initially, 400 subjects were included containing 200 cats and 200 dogs cared between March 2022 and May 2022. Subjects who were not companion animals, could not complete temperature measurement, refused to participate in the study or had an abnormal temperature were excluded. Finally, 369 subjects (180 cats, 189 dogs) exhibiting normal core temperature were used to develop models and initial testing. Later, we further prospectively collected data (30 cats, 30 dogs) between May 2022 and June 2022.

### Data acquirement

**Fig. 2:**
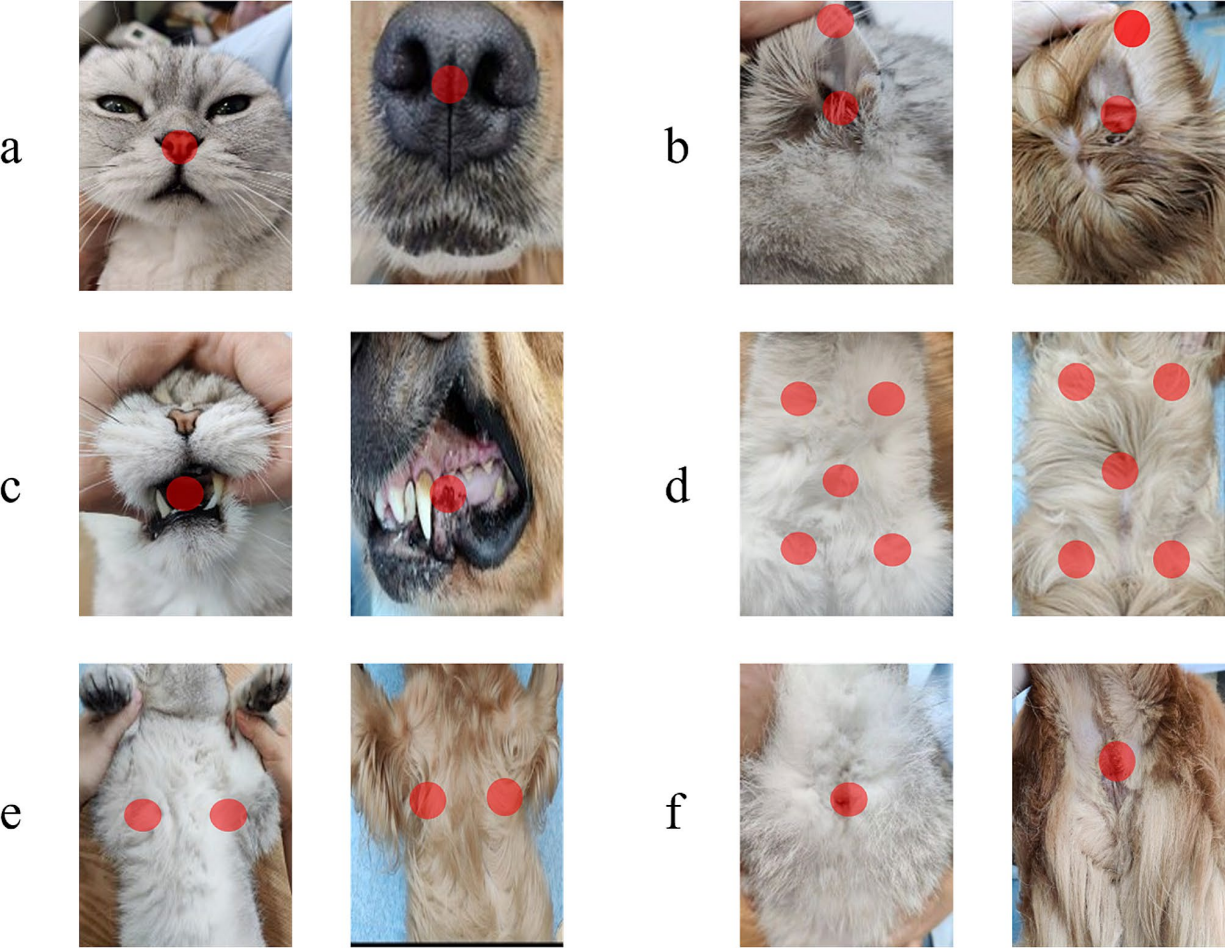
Temperature measurements of surface temperatures, including (**a**) nasal, (**b**) ear, (**c**) oral cavity, (**d**) abdomen, (**e**) axillary, and (**f**) perianal skin. The first and third columns were cats, and the second and forth columns were dogs

We invited four experienced clinicians to form the data team. We explained the study and protocol to the team first before training them with the use of the temperature measuring guns and data recordings. The team was qualified by correctly performing the data acquirements and strictly following the protocol. After practice, the data team was asked to collect data for this study. The RT was measured as the core temperature using a mercury thermometer (Triangle bar type, KeFu, China) and the thermometer was lubricated before use and inserted into the rectum for at least 2 cm and against the rectal wall until a stable peak was reached. To eliminate bias, the anal temperature was measured five times and averaged as the ground truth. Surfaces temperatures were measured using infrared thermometers (KF-HW-011, KeFu, China). In order to identify the best model, we systematically measured temperatures of several body parts including nasal, ears (medial skin of left ear, left ear canal, medial skin of right ear, right ear canal), oral cavity, abdomen (left breast, right breast, central abdomen, lower left abdomen, lower right abdomen), axillary (anterior of left axillary, posterior of left axillary, anterior of right axillary, posterior of right axillary), and perianal, as shown in Fig. 2. The ear temperature was averaged by the temperatures of the four ear areas. The mouth temperature was averaged by the five measurements of oral cavity. The abdomen temperature was the average of the temperatures of the five abdomen areas. The axillary temperature was the average of the temperatures of the four areas. The perianal temperature was the average of the temperatures of five measurements of perianal skin. In result, we obtained six averaged surface temperatures and one room temperature as inputs to machine learning for the core temperature prediction. Both retrospective and prospective studies were conducted at the same clinics and followed the same protocol.

### Machine learning model

In this study, we developed machine learning models for cats and dogs independently, namely dataset of one animal was used to train and evaluate the corresponding model for the animal. In the retrospective study, we first randomly divided the dataset into one train-validation set (80%) and one testing set (20%). To investigate which machine learning model works best in this considered task, we systematically implemented six machine learning models covering commonly used algorithms in studies, including typical linear models, regressors, and regressors based on techniques of boosting and trees. As listed in Table 1, the models were gradient boosting regressor (GBR), support vector regressor (SVR), least absolute shrinkage and selection operator (LASSO), adaptive boosting regressor (ABR), and random forest regressor (RFR). Additionally, we also implemented a multilayer perceptron (MLP) model. In optimizing model performance, we utilized grid search to systematically explore hyperparameter values within manually predefined ranges. Grid search using a selected evaluation metric to assess the performance of machine learning model for each combination within a defined grid. In this instance, a custom loss function, incorporating MAE, MSE, and R2 score with distinct weights, is manually defined for comprehensive model evaluation.

**Table 1:** Machine learning models and hyperparameter settings

No.	Hyperparameter	Description	Value
1	GBR	Gradient boosting regressor	
	Max depth	Maximum depth for each regression estimator	5
	N estimators	Maximum iterations for boosting	70 (cat), 60 (dog)
	$\alpha$	Learning rate used for weight decay	0.02 (cat), 0.05 (dog)
	Criterion	Loss function used for boosting	Absolute error
2	SVR	Support vector regressor	
	Kernel	Kernel function	Radial basis function
	$\gamma$	Kernel coefficient	0.1 (cat), 1 (dog)
	$\lambda$	Regularization coefficient	0.1 (cat), 0.1 (dog)
3	LASSO	Least absolute shrinkage and selection operator	
	$\lambda$	Regularization coefficient	0.5
4	ABR	Adaptive boosting regressor	
	N estimators	Maximum iterations for boosting	45 (cat), 35 (dog)
	$\alpha$	Learning rate used for weight decay	0.05
	Criterion	Loss function to update the weights	Exponential error
5	RFR	Random forest regressor	
	Max depth	Maximum depth for each tree	25 (cat), 40 (dog)
	N estimators	Number of trees in the algorithm	30 (cat), 25 (dog)
	Criterion	Loss function to measure the split quality	Squared error
6	MLP	Multilayer perceptron	
	Activation	Activation function for each neuron	Logistic
	$\alpha$	Learning rate	0.05
	hidden layer sizes	Number of neurons in each hidden layer	15(cat), 20(dog)

We used the 4-fold cross-validation approach in the training-validation process, in which models were trained using three folds (60%) and validated using each of the four folds (20%) iteratively. We select the best-performing model according to the averaged performance in the four cross-validation iterations. The selected model was finally evaluated on the independent testing set. To evaluate the generalization of the selected models, we tested the models in the prospectively collected datasets.

The machine learning models were developed in the programming language Python (3.9.7) using a conventional desktop computer with a central processing unit of Intel Core i5 (4 cores, 2 GHz) and 16 GB of main memory. Opensource libraries of numPy (1.21.2) and Pandas (1.4.1) were utilized for data handling. Scikit-learn (1.0.2) and Keras (2.7.0) were used for implementations of machine learning models and MLP respectively. Matplotlib (3.5.1) was used for visualization.

### Statistical analysis

The performance of the core temperature prediction was evaluated using metrics of mean absolute error (MAE), mean square error (MSE), root mean squared error (RMSE), mean absolute percent error (MAPE). We used RMSE as the main metric. Mathematically, for the ground truth temperature ${t}_{i}$ and its prediction ${\widehat{t}}_{i}$, the metrics were defined as:

$$MAE= \frac{1}{n}\sum _{i=1}^{n}\left|({t}_{i }-{\widehat{t}}_{i})\right|$$

$$MSE= \frac{1}{n}\sum _{i=1}^{n}{\left({t}_{i}- {\widehat{t}}_{i}\right)}^{2}$$

$$MAPE= \frac{100\%}{n}\sum _{i=1}^{n}\left|\frac{{\widehat{t}}_{i}-{t}_{i}}{{t}_{i}}\right|$$

$$RMSE={\left[\frac{1}{n}\sum _{i=1}^{n}{\left({t}_{i}- {\widehat{t}}_{i}\right)}^{2}\right]}^{\frac{1}{2}}$$

## Results

### Subjects characteristics

In the retrospective study, we initially collected 200 subjects for cats and dogs. After data cleaning, the resulting datasets included 180 cats and 189 dogs. In the prospective study, we collected data for 30 cats and 30 dogs. We summarized the subject characteristics including numbers and average temperatures for cats (Table 2) and dogs (Table 3), respectively.

**Table 2:** Characteristics of cats

	Retrospective study					Prospective study
	Total		Train-Validation	Testing		Testing
Number (*n*)	180		144	36		30
Measurements °C (SD*)						
Room	22.5 (1.82)		22.4 (1.83)	22.8 (1.72)		24.1 (1.73)
Nasal	35.7 (1.04)		35.7 (1.02)	35.9 (1.12)		34.5 (0.41)
Ear	36.8 (0.88)		36.8 (0.86)	37.0 (0.93)		34.6 (0.51)
Oral cavity	35.5 (0.61)		35.5 (0.61)	35.7 (0.58)		35.6 (0.48)
Abdomen	35.2 (0.60)		35.2 (0.60)	35.2 (0.60)		36.0 (0.26)
Axillary	34.5 (0.85)		34.5 (0.86)	34.5 (0.81)		36.0 (0.30)
Perianal	35.7 (0.64)		35.7 (0.66)	35.7 (0.54)		36.1 (0.29)
Core (anal)	38.5 (0.21)		38.5 (0.20)	38.6 (0.25)		38.5 (0.14)

*SD: Standard Deviation

**Table 3:** Characteristics of dogs

	Retrospective study				Prospective study
	Total	Train-Validation	Testing		Testing
Number (*n*)	189	151	38		30
Measurements °C (SD*)					
Room	22.5 (1.89)	22.4 (1.86)	22.6 (2.02)		23.4 (1.68)
Nasal	33.7 (0.68)	33.7 (0.69)	33.7 (0.63)		34.3 (0.48)
Ear	34.6 (0.58)	34.7 (0.62)	34.5 (0.40)		34.2 (0.43)
Oral cavity	35.5 (0.38)	35.5 (0.41)	35.5 (0.23)		35.1 (0.32)
Abdomen	34.2 (0.64)	34.3 (0.68)	34.0 (0.39)		35.1 (0.54)
Axillary	35.2 (0.45)	35.2 (0.46)	35.1 (0.41)		36.0 (0.25)
Perianal	35.7 (0.43)	35.7 (0.41)	35.5 (0.48)		36.0 (0.27)
Core (rectal)	38.5 (0.20)	38.5 (0.21)	38.5 (0.14)		38.5 (0.14)

*SD: Standard Deviation

### Performance of machine learning

**Fig. 3:**
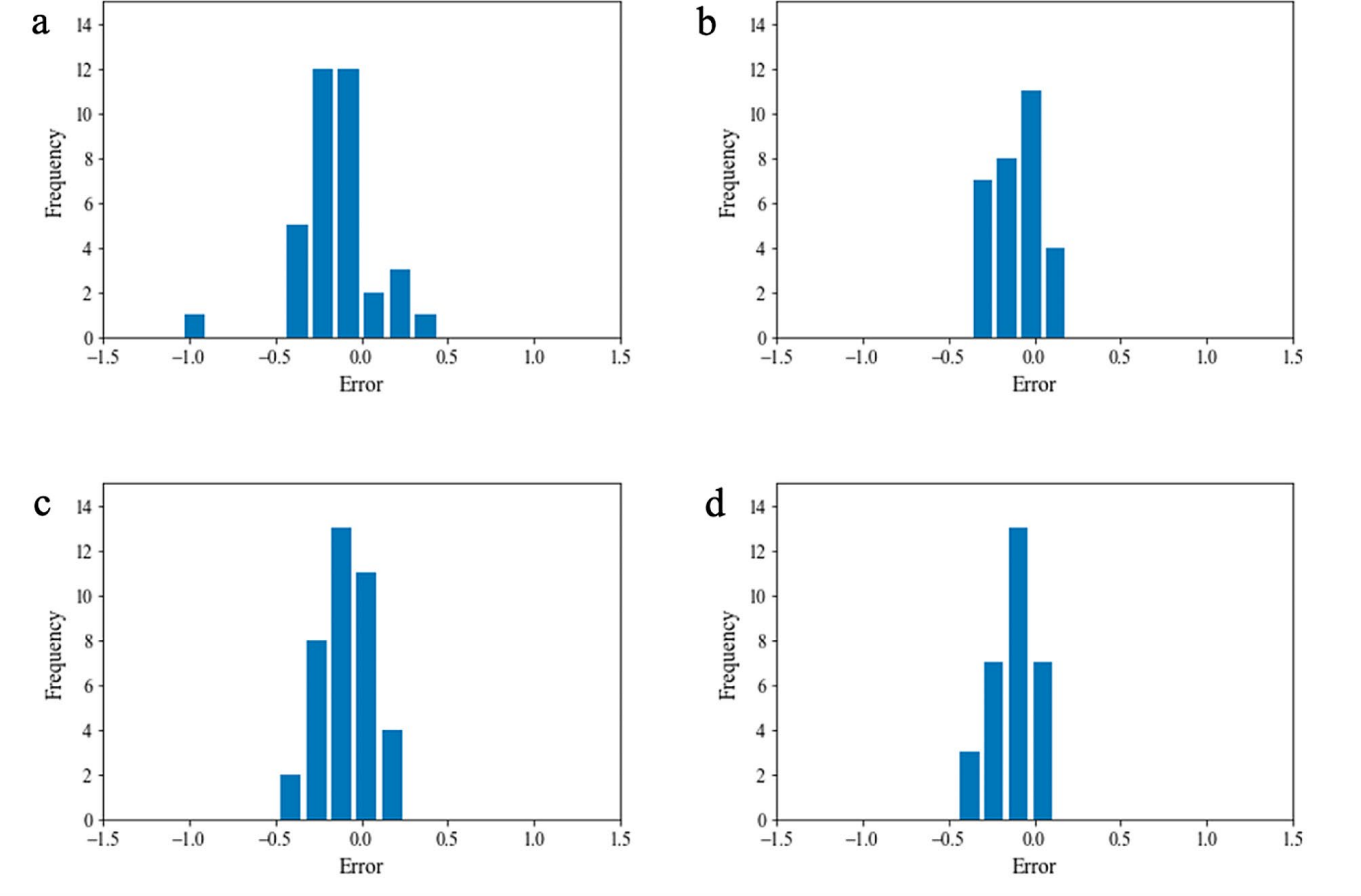
Histograms of prediction errors for the selected models in retrospective and prospective studies. The first row were cats in retrospective (**a**) and prospective (**b**) studies, and the second row were dogs in retrospective (**c**) and prospective (**d**) studies

In this study, we used the cross-validation approach to train and evaluated multiple machine learning methods in both respective and prospective studies. We first reported the performance obtained in the cross-validation of the retrospective study. As shown in Table 4, ABR and SVR obtained relatively optimal performance with RMSE of 0.21 and 0.21 for cats and dogs, respectively. Therefore, we selected ABR as the chosen model for cats and SVR for dogs. We evaluated the performance of the chosen models first in the retrospective testing set and later in the prospective testing set. The final results were summarized in Table 5. We found that the models could accurately predict the core temperatures for both cats and dogs with satisfying performance. The values of RMSE for cats in respective and prospective studies were 0.25 and 0.15, respectively. The values of RMSE for dogs in respective and prospective studies were 0.15 and 0.14, respectively. In Fig. 3, we illustrated the histograms of prediction errors in both studies. As shown, most predictions fall around zero with small deviations, indicating good accuracies. In order to evaluate the model’s performance across different cohorts, we also counted the number of cats and dogs that had an absolute error (AE) less than the corresponding SD in each cohort. In the retrospective cohort, 21 cats and 27 dogs exhibited AE below the retrospective SD. Similarly, in the prospective cohort, 17 cats and 22 dogs demonstrated AE below the prospective SD. These numbers also show good accuracies. We calculated quantiles of the RMSE for cats and dogs to further evaluate the models’ predictive performance. The RMSE quantiles for retrospective cats were found to be [0.03, 0.06, 0.11] for the 25th, 50th, and 75th percentiles of the lowest absolute errors, respectively. Similarly, for retrospective dogs, the RMSE quantiles were [0.03, 0.07, 0.08]. In the prospective cohort, the RMSE quantiles for cats were [0.03, 0.06, 0.10], and for dogs, the RMSE quantiles were [0.02, 0.04, 0.07]. The results show the models have good performance at different levels of prediction accuracy.

**Table 4:** Performance of models in the cross-validation

Model	MAE			MSE			MAPE			RMSE	
	Cat	Dog		Cat	Dog		Cat	Dog		Cat	Dog
SVR	0.15	0.15		0.04	0.05		0.40%	0.38%		0.21	0.21
ABR	0.15	0.15		0.04	0.05		0.40%	0.40%		0.21	0.21
LASSO	0.15	0.15		0.04	0.05		0.38%	0.38%		0.20	0.21
GBR	0.16	0.16		0.04	0.06		0.40%	0.42%		0.21	0.23
LR	0.16	0.15		0.04	0.05		0.41%	0.39%		0.21	0.21
RFR	0.17	0.17		0.05	0.05		0.45%	0.44%		0.22	0.23
MLP	0.22	0.23		0.07	0.07		0.56%	0.58%		0.27	0.27

**Table 5:** Performance of the selected models in the retrospective and prospective testing sets

Study	Animal	MAE	MSE	MAPE	RMSE
Retrospective	Cat	0.17	0.06	0.44%	0.25
	Dog	0.12	0.02	0.31%	0.15
Prospective	Cat	0.12	0.02	0.31%	0.15
	Dog	0.09	0.02	0.24%	0.14

## Discussion

As the closest companion animals to human beings, the diseases of cats and dogs have a great impact on human physical and mental health [5]. As an important index of core body temperature, the RT has important guiding significance in the diagnosis and treatment of pet diseases. However, traditional RT measurement methods have some disadvantages, such as poor tolerance, the influence of intestinal contents, and lag, making it difficult to measure the core temperature of animals quickly and accurately. This study proposed an alternative and simple method to predict core temperatures for companion animals, based on body surface temperatures using machine learning. The results demonstrated the accuracies were encouraging.

In the existing literature, one study developed models to predict RT, skin-surface temperatures, and hair-coat surface temperatures for livestock like piglets using lamps as supplementary heats [24]. In another study, rectal temperatures of rabbits were predicted using measurements obtained with advanced infrared cameras [23]. There were also reports for human infants, among them, Lyra et al. combined deep learning-based algorithms and camera modalities to real-time monitor the temperature of neonates [25]; Yaeger et al. developed a natural language processing algorithm to identify febrile infants [26]; Asano et al. applied a semantic segmentation method to thermal images, which makes it possible to monitor the temperature distribution over the whole body of infants [27]. Different to those approaches in predicting core temperatures, our method focused on companion animals using convenient operations and equipment. Moreover, we systematically investigated a broad range of machine learning models and considered more body surface parts.

Our results suggested that the proposed method demonstrated several advantages in determining the core temperatures of human-companion animals. First, by measuring the surface temperatures, our non-invasive, contactless approach was friendly to shy animals than the traditional method of invasively measuring anal temperatures. Professional clinicians, as well as animal owners, could easily master the simple measuring techniques in no time without the need for complicated training. Moreover, our method used widely available and affordable temperature measuring guns, making it possible for rapid adoption. Therefore, our approach had promise in assisting diagnosis in animal clinics and daily animal health monitoring in households. Altogether, the present study added new evidence to the line of studies using machine learning to predict core temperatures of animals, meanwhile providing valuable implications for the prediction of the core temperatures of human infants.

This study also had limitations. First, this study is a single-center study with limited resources. In the future, a larger-scale of multicenter study could strengthen the dataset. Second, we only included cats and dogs in this study, other human-companion animals were not studied. The potential usefulness and generalization capability were unknown and worth further investigations in other human-companion animals. Third, due to limited data, we did not include the age, sex, breed, feeding situation, health status, hair condition of animals, and other factors like humidity, day-night timing of measurements, and seasons. Future work could consider these potential influential variables. Four, as an initial study, we measured the temperatures of several body parts. To facilitate the adoption of the proposed method, we need to reach a balance of convenience and acceptable accuracy. Namely, future investigations should identify the optimal combination with the least measurements of body parts for easier uses in practice. Last but not least, the present work was only a primitive model. It’s worth implementing this model as a working system to further evaluate the model and cumulate incoming data for model retrains.

## Conclusions

In this study, we proposed a machine learning method to accurately predict the core temperatures of cats and dogs using measurements of other surface body parts. We conducted a retrospective study using the first dataset to identify and develop the best-performing model. Subsequently, a prospective study was conducted using the second dataset to validate the selected model’s performance on new, unseen data and to gain insights into long-term trends. We implemented several machine learning models and trained using cross-validation.

## Data Availability

The data used in this study is available from the corresponding author on reasonable request.

## Abbreviations

RT: Rectal Temperature
AI: Artificial Intelligence
ML: Machine Learning
MLP: Multilayer Perceptron
GBR: Gradient Boosting Regressor
SVR: Support Vector Regressor
LASSO: Least Absolute Shrinkage and Selection Operator
ABR: Adaptive Boosting Regressor
RFR: Random Forest Regressor
MAE: Mean Absolute Error
MSE: Mean Square Error
MAPE: Mean Absolute Percent Error
RMSE: Root Mean Squared Error
AE: Absolute Error
